# Human Challenge Pilot Study with *Cyclospora cayetanensis*

**DOI:** 10.3201/eid1004.030356

**Published:** 2004-04

**Authors:** Edith M. Alfano-Sobsey, Mark L. Eberhard, John R. Seed, David J. Weber, Kimberly Y. Won, Eva K. Nace, Christine L. Moe

**Affiliations:** * Office of Public Health Preparedness and Response, Raleigh, North Carolina, USA; † Centers for Disease Control and Prevention, Atlanta, Georgia, USA; ‡ University of North Carolina, Chapel Hill, North Carolina, USA; § Emory University, Atlanta, Georgia, USA

**Keywords:** Cyclospora cayetanensis, Infectivity, Human challenge study

## Abstract

We describe a pilot study that attempted to infect human volunteers with *Cyclospora cayetanensis*. Seven healthy volunteers ingested an inoculum of *Cyclospora* oocysts (approximately 200–49,000 oocysts). The volunteers did not experience symptoms of gastroenteritis, and no oocysts were detected in any stool samples during the 16 weeks volunteers were monitored.

*Cyclospora cayetanensis* is a protozoan parasite that may cause gastroenteritis with prolonged, intermittent diarrhea in humans. Characterizatioin and magnitudes of risk factors associated with food and water consumption are unclear because the dose-response relationship and other host-parasite factors for infection with *Cyclospora* are unknown. To characterize infectivity, we performed a study in which inocula of *Cyclospora* oocysts were administered to human volunteers.

## The Study

Stool samples containing high concentrations of *Cyclospora* oocysts and serum specimens were collected from persons with cyclosporiasis in Haiti and the United States. Informed consent was obtained from persons providing the specimens. The Centers for Disease Control and Prevention (CDC) institutional review board (IRB) reviewed specimen collection, informed consent, and patient counseling procedures. Stool specimens were homogenized with water and sieved through cheesecloth. Since *Cyclospora* oocysts must form sporocysts outside the human host before becoming infectious, the filtrate was resuspended in potassium dichromate (2.5%) and shaken for about 3 weeks at room temperature to induce sporulation. After sporulation (67%–94% of oocysts sporulated), samples were stored at room temperature until further processing (2–3 months)**.** Suspensions with the highest oocyst counts were purified and concentrated by sucrose and cesium chloride gradients (*1*). See the Table for additional inoculum treatment conditions.

**Table:** Inoculum treament and challenge conditions from the *Cyclospora* human challenge study^a^

Volun-teer	Oocysts source (no.)	Oocysts ingested, mean ± SD (% sporulated)	Inoculum disinfection^b,c^	Storage medium until dosing	Total storage (mo)^d^	Inoculum ingestion medium and conditions
1	Haiti (3)	647 ± 183 (82)	Bleach and thiosulfate (pre-storage)	Sterile water	6+	Sterile water, then light lunch
2	Haiti (3)	203 ± 22 (69)	Bleach and thiosulfate (pre-storage)	Sterile water	7+	Sterile water, then light lunch
3	Haiti (1)	884 ± 22 (96)	Bleach and thiosulfate (pre-storage)	Sterile water	10	Sterile water, then light lunch
4	MO, USA (1)	4,851 ± 545 (71)	Bleach only; wash on dosing day	Sterile PBS	6+	Sterile water, with a chicken salad meal
5	MO, USA (1)	1,815 ± 249 (67)	Thiosulfate only; wash on dosing day	Sterile PBS	7	Sterile water, with a chicken salad meal
6	MO, USA (1)	4,916 ± 1,153 (67)	Bleach and thiosulfate (pre-storage)	Sterile PBS	7+	Sterile water, with a chicken salad meal
7	GA, USA (1)	48,884 ± 15,345 (94)	Bleach and thiosulfate 3 days predosing	2.5% dichromate	5	Raspberries, then light lunch

^a^MO, Missouri; GA, Georgia; PBS, phosphate-buffered saline. ^b^Household bleach (5.25% sodium hypochlorite) was used for disinfection, and sodium thiosulfate (0.01%–0.1%) was used to inactivate residual bleach in the inocula. ^c^For safety testing laboratory details, contact corresponding author. ^d^Time in storage from stool collection until volunteer dosing date, includes time oocysts were exposed to potassium dichromate in stool until sporulation plus the time the oocysts were in storage media after they were extracted from the stool specimens.

Each candidate inoculum was tested for *Salmonella* spp., *Shigella* spp., *Campylobacter* spp., *Yersinia* spp., *Mycobacterium* spp., *Escherichia coli* O157:H7, enteroviruses, *Hepatitis A virus* (HAV), *Herpesvirus*, *Cytomegalovirus*, *Coronavirus*, *Astrovirus*, *Rotavirus*, *Norovirus*, *Adenovirus*, HIV, *Clostridium difficile* toxin, enterotoxin, and intestinal parasites (data not shown). Serum specimens from the donors of *Cyclospora*-positive stools were tested for serum markers of HIV, HAV, *Hepatitis B virus*, and *Taenia solium*. If serum from a *Cyclospora*-positive stool donor tested positive for any of the above, oocysts from that person were not used. Candidate inocula in which none of these pathogens or toxins were found were used in the human challenge study. This safety-testing protocol was reviewed and approved by both the University of North Carolina School of Medicine IRB and the CDC IRB. Cell culture infectivity assays and animal models were not available to determine the infectivity or viability of the oocysts. Attempts were made to assess the viability of the inoculum by observing sporozoite motility after excystation of the oocysts by different methods. However, these methods did not yield a sufficient number of motile sporozoites to measure viability.

The study was conducted at the General Clin ical Research Center at the University of North Carolina Hospital, Chapel Hill, NC. Inclusion and exclusion criteria determined whether a person was eligible to participate in the study. Healthy volunteers were recruited from the University of North Carolina (UNC), Chapel Hill, NC, and the surrounding community. Before enrollment, each candidate received a medical evaluation, and preinoculation serum specimens were collected and archived. The seven study participants comprised four women and three men; three were white, and four were African American. The median age was 26.

After ingesting the inoculum, volunteers were asked to collect all stool specimens for 4 weeks and one specimen a week at weeks 5, 6, 8, and 16. They were also asked to keep a daily record of physical symptoms and the time of each stool passage. In addition, volunteers provided blood and saliva specimens weekly for 6 weeks and at weeks 8 and 16 postdosing. Study outcome measures were a) shedding *Cyclospora* oocysts in stool; b) frequency, weight, color, and consistency of stool; and c) clinical symptoms of gastroenteritis: diarrhea (>3 stools in 24 hours), nausea, vomiting, abdominal pain, myalgia, headache, fever, chills, or fatigue.

Stools were examined to detect oocysts at UNC Chapel Hill. All stool specimens were concentrated by using the formalin-ethyl acetate concentration procedure routinely used to examine ova and parasites in stool specimens (*2*). In addition, to increase the sensitivity of detection, all stools from the first 2 weeks postdosing were concentrated by a sucrose floatation procedure (*1*). Aliquots of the concentrated sample from each procedure were examined microscopically by using a wet mount preparation (*2*), and 87% of these concentrates were confirmed by a second laboratory at CDC. Criteria to identify *Cyclospora* oocysts were based on size, morphologic characteristics, and ability of the oocysts to autofluoresce under epifluorescence (*3*).

Inoculum treatment and challenge conditions of this study are described in the Table. Numbers of stools examined per volunteer ranged from 19 to 40; no oocysts were detected in any of the stool samples. Volunteer 1 experienced a brief episode of abdominal cramps on day 7 postdosing. This volunteer attributed the symptom to possible dehydration due to strenuous activity performed in the heat that day. Volunteer 5 produced four loose stools on day 10 postdosing but reported feeling well. This volunteer also had a mildly elevated leukocyte count (15.8 x 10^9^/L; normal range 4.5–11.0 x 10^9^/L) on day 13 postdosing. However, this volunteer had a mildly elevated baseline leukocyte count (13.8 x 10^9^/L) 14 days before dosing. Overall, no conclusive evidence based on clinical or parasitologic diagnostic procedures showed that any volunteers became infected.

## Conclusions

Cyclosporiasis continues to be a difficult emerging infectious disease to understand. Our results are consistent with other researchers’ inability to establish *C. cayetanensis* infection in a wide variety of animal models (*4*). Given these results, questions relating to host susceptibility and risk factors for infection with *Cyclospora*, the biology of *C.* *cayetanensis*, survival conditions for *C.* *cayetanensis* in vitro and in vivo, and factors that allow *Cyclospora* to become infectious in the environment need further study.

Host susceptibility and risk factors for infection are always a consideration when evaluating host response to pathogen exposure. Epidemiologic data suggest that immunity may develop to *C.* *cayetanensis* in areas where cyclosporiasis is endemic and that the disease is more severe in naïve populations (*5*). Persons affected in foodborne outbreaks of cyclosporiasis in North America were mostly adults who experienced prolonged symptomatic gastroenteritis, and median food-specific attack rates were high (*6*,*7*). In this study, only healthy adult volunteers (22–53 years of age) were recruited from areas in which cyclosporiasis is not known to be endemic. Therefore, although the number of volunteers in this study was small (N = 7), epidemiologic data suggest that host susceptibility factors did not substantially contribute to inability of the inocula to cause infection in the volunteers at the doses administered.

Virulence and characteristics of *Cyclospora* necessary to infect human hosts are unknown. Nucleotide sequence variability in the first internal transcribed spacer regions within *C. cayetanensis* from different geographic origins has been observed and suggests the existence of multiple strains (*8*,*9*). In addition, data from *Cryptosporidium* human volunteer studies demonstrated that the 50% infectious dose (ID_50_) differed (from 9 to 1,042 oocysts), depending on the isolate used in the study (*10*). For these reasons*,* we attempted to vary the inocula by selecting oocysts from persons in different geographic regions (Haiti, Missouri, and Georgia) and increasing the numbers of oocysts ingested by volunteers (from <1,000 oocysts to approximately 49,000) during the course of this study. Thus, differences in virulence characteristics of *C. cayetanensis* isolates appear not to have been a major factor in failing to establish infection.

All oocysts in stool samples in this study were stored in potassium dichromate (2.5%), and most of the final inoculum preparations were disinfected with bleach (5.25%). *Cryptosporidium* *parvum* has been stored in 2.5% potassium dichromate (for <6 weeks to >12 weeks) and remained infectious in human volunteers, cell culture, and animals (*11*,*12*). Also, baboons inoculated with oocysts never exposed to potassium dichromate were not infected with *Cyclospora* (M. Eberhard, unpub. data). Other parasites of genera related to *Cyclospora* (*Cryptosporidium*, *Eimeria* spp., *Toxoplasma gondii*, sporocysts of *Sarcocystis* spp.) have been shown to resist high levels of bleach (*13*–*15*). However, the effects of potassium dichromate and bleach on the *Cyclospora* oocysts used in this study are unknown, since methods to evaluate infectivity and viability were not available.

Naturally occurring *Cyclospora* oocysts may survive for extended periods in the environment, given the marked seasonality of infection in areas where the disease is endemic (*6*). However, many questions remain about the triggers and conditions necessary for *Cyclospora* oocysts to survive and become infectious in the environment. Given the results of this study, conditions necessary for *Cyclospora* to become infectious were probably not achieved in preparing and storing the oocysts. Future studies are necessary to examine individual and combined effects of temperature, humidity, storage media, and disinfection on the survival, viability, and infectivity of stored *Cyclospora* oocysts. These studies would help determine optimal conditions to stimulate sporulation and maintain infectivity of oocysts in vitro over time. However, such studies will not be possible until suitable cell culture systems or animal host models for cyclosporiasis are developed.
